# High Altitude Bird Migration at Temperate Latitudes: A Synoptic Perspective on Wind Assistance

**DOI:** 10.1371/journal.pone.0052300

**Published:** 2013-01-03

**Authors:** Adriaan M. Dokter, Judy Shamoun-Baranes, Michael U. Kemp, Sander Tijm, Iwan Holleman

**Affiliations:** 1 Institute for Biodiversity and Ecosystem Dynamics, University of Amsterdam, Amsterdam, The Netherlands; 2 Royal Netherlands Meteorological Institute, De Bilt, The Netherlands; 3 Radboud University Nijmegen, Nijmegen, The Netherlands; University of Milan, Italy

## Abstract

At temperate latitudes the synoptic patterns of bird migration are strongly structured by the presence of cyclones and anticyclones, both in the horizontal and altitudinal dimensions. In certain synoptic conditions, birds may efficiently cross regions with opposing surface wind by choosing a higher flight altitude with more favourable wind. We observed migratory passerines at mid-latitudes that selected high altitude wind optima on particular nights, leading to the formation of structured migration layers at varying altitude up to 3 km. Using long-term vertical profiling of bird migration by C-band Doppler radar in the Netherlands, we find that such migration layers occur nearly exclusively during spring migration in the presence of a high-pressure system. A conceptual analytic framework providing insight into the synoptic patterns of wind assistance for migrants that includes the altitudinal dimension has so far been lacking. We present a simple model for a baroclinic atmosphere that relates vertical profiles of wind assistance to the pressure and temperature patterns occurring at temperate latitudes. We show how the magnitude and direction of the large scale horizontal temperature gradient affects the relative gain in wind assistance that migrants obtain through ascending. Temperature gradients typical for northerly high-pressure systems in spring are shown to cause high altitude wind optima in the easterly sectors of anticyclones, thereby explaining the frequent observations of high altitude migration in these synoptic conditions. Given the recurring synoptic arrangements of pressure systems across temperate continents, the opportunities for exploiting high altitude wind will differ between flyways, for example between easterly and westerly oceanic coasts.

## Introduction

Temperate latitudes show a high spatial and temporal variability in weather, related to the frequent passage of high and low pressure systems [1], [2]. These pressure systems have been shown to play a ubiquitous role in structuring the temporal and spatial patterns of both bird and insect migration. Migration can take place in a wide variety of wind conditions [3]–[5], but peak movements are found primarily in following wind conditions [2], [3], [5]–[9] (and references therein). Expert classification of weather systems has been used to deduce the preferred sectors of cyclones and anticyclones for migration [2], [6], [10]. For example, during northward spring migration, migrants will prefer the easterly sectors of low-pressure cyclones and westerly sectors of high-pressure anticyclones (as well as their calm centres), where they can exploit the benefits of a free ride on the wind.

Wind conditions may change considerably with altitude, therefore migratory birds may be able to control the atmospheric conditions they experience by selecting a particular flight altitude. The effect of altitudinal changes of wind on flight altitude has been primarily studied in the trade wind zone of the northern hemisphere (0–30° latitude) [11]–[13] and adjacent areas [14]. The general circulation in these areas is characterised by two opposing trade winds blowing towards the equator at low altitude and away from the equator at high altitude. In spring, migrating passerines were shown to avoid the opposing low-level trade and ascend into the supportive trade at altitudes up to 3 km above ground [12], [13], thus forming a high-altitude migration layer. Wind assistance was identified as a key factor determining flight altitude, with various physiological constraints on temperature, humidity and oxygen pressure being of limited importance only [13], [15]–[17].

At temperate latitudes the atmosphere may show strong changes in wind with altitude as well [1], and similar to the trade wind zone birds can be expected to use high-altitude migration to efficiently cross regions with opposing surface wind. It is however conceptually difficult to infer which sectors of cyclones and anticyclones have favourable winds for high versus low altitude migration, given their relative positioning across a continent. A simple analytical framework providing a synoptic perspective on how wind assistance changes with flight altitude has so far been lacking, and the first aim of this paper is to fill this gap. Besides bird migration, such a framework may be equally informative for understanding wind effects on other aerial migrants, such as the migration and layering of insects [18], [19]. A second aim of this paper is to identify the meteorological conditions in which high altitude migration, specifically layering, occurs at mid-latitudes and to test whether these observations fit within the predicted synoptic patterns for flight altitude.

## Methods

### Theory of mid-latitude wind profiles and wind assistance

For a synoptic perspective on flight altitude selection by birds, it is instructive to introduce the properties of vertical wind changes in relation to the (anti)cyclonic weather systems found at mid-latitudes. Synoptic patterns of wind, pressure and temperature are ultimately linked through the fundamental laws of fluid mechanics and thermodynamics, which govern the motions of the atmosphere. We may use these laws to describe altitudinal changes of wind in terms of the synoptic patterns of temperature, which in our context can be more insightful than the more familiar approach of describing wind in terms of atmospheric pressure patterns.

Air flow in mid-latitude atmospheres generally satisfies two good approximations: (1) hydrostatic balance, i.e. pressure is given by the weight of the air column above, and (2) geostrophic balance, i.e. the pressure gradient force (acting perpendicular to isobars) is exactly cancelled by the Coriolis force (acting perpendicularly to the direction of flow) [1]. In the process of changing flight altitude from altitude 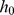 to an altitude 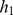, a bird could add the difference vector of wind between these two flight altitudes, written as 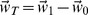, to the wind experienced at the take-off altitude 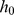. For the geostrophic component 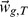 of this difference vector (i.e. neglecting turbulence induced by the earth's surface, see Text S1 of the supporting information for further details), it can be shown that in an ideal gas atmosphere
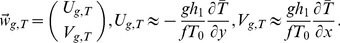
(1)Here, 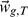 has been written in its terms of its individual components U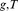 (towards east, along Cartesian×coordinate) and V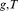 (towards north, along Cartesian y coordinate). 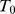 equals the temperature at the take-off altitude, 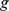 the gravitational acceleration and 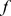 the Coriolis parameter. The overbar on the temperature derivatives and on the mean temperature gradient of the air layer 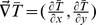 denotes a vertical average over the layer from 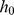 to 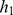.

The above expression for the (geostrophic) wind difference 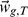 between two altitudes is called the *thermal wind* [1]. The equation shows that the change in horizontal wind with altitude can be expressed in a very simple form in terms of the large-scale geographic (horizontal) patterns of surrounding temperature, i.e. the temperature gradient 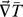. In particular, the thermal wind 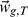 always blows perpendicular to the temperature gradient 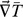. The thermal wind equation can thus be used to understand vertical changes of wind in terms of large scale temperature patterns found at mid-latitudes. Whether ascending to a given altitude is beneficial for a bird in terms of wind, will depend on whether addition of the corresponding thermal wind vector to the surface wind improves a bird's perceived assistance of wind and efficiency of transport [20].

**Figure 1:**
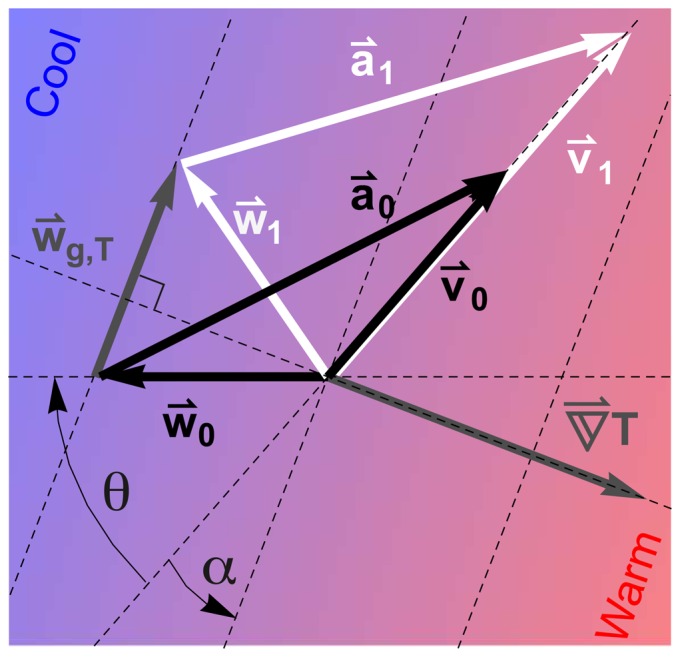
Triangle of velocities. Air, wind and ground speed vectors (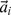, 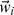, 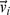) for a fully compensating bird at ground level (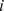 = 0, black arrows) and at a given altitude 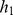 (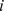 = 1, white arrows). The surface and high altitude geostrophic winds 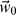, 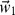 are related via the thermal wind vector: 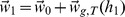. Angle 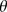 equals the wind direction with respect to the preferred migratory direction - 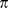, i.e. 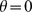 denotes a full head wind. Angle 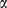 equals direction of the thermal wind with respect to the preferred direction, i.e. the angle between isotherm and preferred direction. Angles 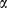 and 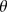 are defined positive clockwise.

In order to determine which wind conditions are beneficial to a bird, assumptions must be made about its flight behaviour. How birds respond to wind to migrate most efficiently is a research topic in itself [10], [21] and beyond the scope of this paper. As a model of reference, we will assume that birds have a fixed airspeed 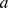 and fully compensate for wind drift by adjusting their heading (e.g. see [13], [20], [22] for further details). Ground speed 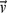, wind speed 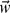 and self-propelled airspeed 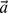 are thus related according to 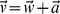 and birds are assumed to maintain a ground speed vector 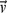 aligned with their preferred migration direction in all circumstances. In Figure 1 we have illustrated the triangle of velocities made up by a bird's ground speed and air speed vectors and the wind speed vector both at the ground 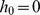 (black arrows) and at a given altitude 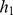 (white arrows). The surface and high altitude winds 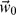, 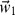 are related via the thermal wind vector 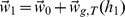. Using this relation and simple trigonometry we can express the resultant ground speeds as follows:
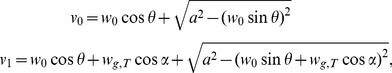
(2)where 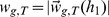, 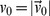, 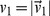, 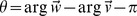 the angle of the wind with the bird's preferred direction (defining 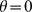 as a full head wind) and 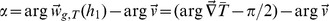 the angle between the mean isotherm within the traversed air column and the bird's preferred direction (see Figure 1). Taking the argument of a vector, i.e. its directional angle, is denoted by ‘arg’.

To quantify the favourability of wind conditions for a migrating bird, we may adopt a commonly used formulation of wind assistance (WA) (e.g. see [13], [20], [22] for further details), defined as the achieved ground speed in a full compensation strategy minus the airspeed maintained by self-propelled flight: 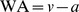. According to this definition the change in wind assistance 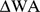 through ascending from 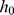 to 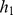 equals
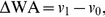
(3)which equals the gain in ground speed through ascending. It should be clear to the reader that field studies have shown that there may be variation among species in airspeed and preferred direction [20] (and references therein). Also, birds do not necessarily achieve nor seek perfect wind compensation, but often tolerate wind drift [10], [21] (and references therein), with recent studies suggesting partial drift compensation by nocturnal passerines at temperate latitudes [23]. Wind assistance and travel speeds as calculated above should therefore be interpreted with care. For a bird that accepts drift, cross winds may have a less negative impact on the perceived wind assistance than predicted by equation 3 [20]. Analogously, for thermal winds that can reduce cross wind (i.e. in the case of ascents of birds flying parallel to 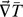), the improvement in WA may be overestimated. Earlier studies have shown that equation 3 is useful as a qualitative indicator of the favourability of wind dependent on both tail and cross winds (e.g. see [13], [20], [22]), and we will use it accordingly. The main conclusions of this paper will not depend critically on the definition of wind assistance or assumed drift compensation strategy, similarly other measures of wind assistance could be used.

### Radar measurements of bird density

We used methods described by Dokter et al. [24] to derive altitude profiles of bird density 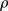 (birds/km^3^) and average groundspeed (m/s) every five minutes from a C-band Doppler weather-radar located in De Bilt, the Netherlands (52.11°N 5.18°E) during spring (1 February–31 May) and autumn (1 August–30 November) of 2008 and 2009. The radar antenna is located on a tower 42 m above ground level (44 m above mean sea level). Bird profiles were determined from 0.4 to 4 km in altitude bins of 200 meter. Altitudes used throughout are above ground level [AGL]. Data in the lowest two altitude bins (up to 0.4 km) were excluded from the analysis, to discard contaminations of ground clutter. For the analysis of bird density profiles we made a strict selection for nights in which no precipitation was observed within the radar volume (precipitation is expected to have pronounced effects on bird flight altitudes [25], however this is beyond the scope of the current study).

Birds need some time to settle at a flight level after take-off (see e.g. [15]), we therefore took the bird density altitude profile at 2.5 hours after sunset as the representative profile. Nights were included in the analysis when the altitude-integrated bird density in the air column exceeded 10 birds/km^2^. Nights with migration above 800 m above the detection limit of 1 bird/km^3^, but with an altitude-integrated bird density below 10 birds/km^2^, were also included in the dataset (note that a height-integration of a volumetric density gives a surface density, which has a dimensionality km^−2^ instead of km^−3^). The selection of nights thus did not treat low and higher altitude migration equivalently: in the regime of low bird density, higher altitude migration nights were included more often. This is because the presence of birds can be reliably determined at low densities at higher flight altitudes, but not in lowest strata due to possible contamination with clutter and insect scattering [24].

Out of 241 spring nights, 102 nights were free of precipitation, of which 75 nights contained migration. Out of 240 autumn nights, 71 were free of precipitation, of which 66 nights contained migration. The total dataset thus consisted of 141 migration nights that were precipitation free.

### Meteorological data

We retrieved meteorological data from the high-resolution atmospheric model Hirlam [26], using data from the grid point nearest the centre of the De Bilt radar (33 km east at 5.64°E 52.02°N). These data had a spatial resolution of 0.1°×0.1°, temporal resolution of one hour, and were discretised vertically at fixed pressure levels separated by not more than 20 mb.

To quantify the favourability of wind conditions we used the formulation of wind assistance (WA) of equations 2 and 3. In autumn we assumed 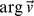 = 221° clockwise from north, the mean migratory direction in autumn as observed by bird radar at our study site [24], similar to other radar observations throughout Europe cf. [27], [28]. In spring we assumed 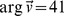
°, as observed by a bird radar in Trappes in northern France [24], to our knowledge the bird radar study conducted nearest to our study site with published ground speeds and flight directions based on individual tracks. Because passerines dominate nocturnal migration over Europe [29], we set the birds' airspeed 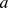 to 12 m/s, which is a representative mean for airspeeds found in many migrating passerine species [30], [31]. In this definition WA varies with the wind vector 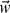 only.

All layering events of 2008 and 2009 were classified as occurring under influence of either a low or high-pressure system, following the synoptic analysis at 500 hPa pressure level prepared daily by the Royal Dutch Meteorological Institute [32], [33].

### Definition of layering and wind optima

**Figure 2:**
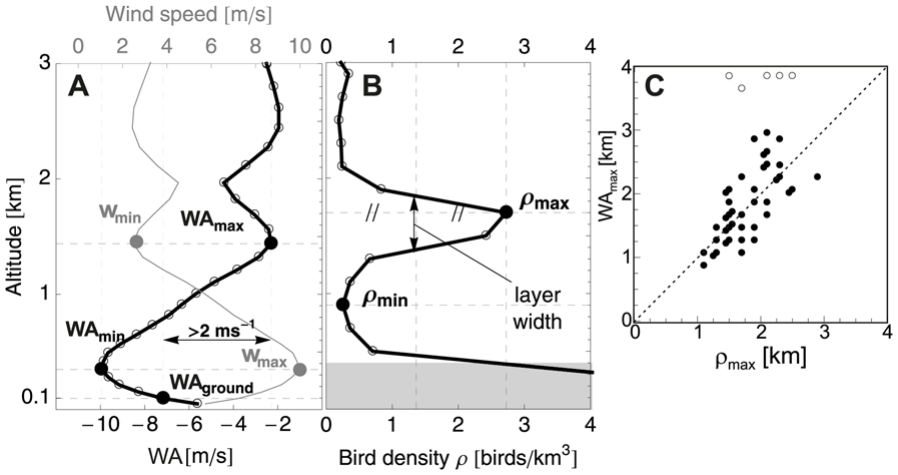
Definition of layering and wind extrema. A: Wind assistance profile (black; WA), wind speed profile (grey; v) for 19 May 2008 22:00 UTC. WA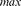 equals the first WA maximum reached by ascending birds that is at least 2 ms^−1^ more supportive than the surface wind WA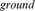 taken at 100 m AGL. B: the corresponding bird density profile (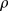) with a layer at altitude 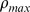. C: Altitude of the layer 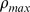 versus altitude of the wind optimum WA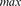.

We concentrated our analysis on migration events where high altitude migration is preferred over low altitude migration, which leads to formation of migration layers. We define layering as a bird density profile that reaches a density maximum at certain altitude *above* ground level, in which the peak density was at least a factor 2 higher than the density minima at lower altitude (bird density 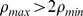, see Figure 2). The lowest possible layering altitude was in the 600–800 m AGL bin, as we leave out the lowest 400 m from the analysis. The layer altitude was defined as the altitude of the modal (highest) bird density (altitude 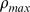, see Figure 2). The nights excluded due to precipitation did not contain layers, i.e. all layering events of 2008 and 2009 were contained in the analysed dataset.

Wind assistance maxima and minima were identified as points where the first derivative of WA with respect to altitude was zero (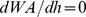). Small-amplitude WA extrema were neglected by requiring that the amplitude of the extremum directly below differed by at least 1 ms^−1^, a procedure used previously in the rejection of small maxima and minima in wind speed profiles by Baas et al. [34]. The wind optimum WA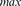 - used as a predictor of layering altitude - was the first WA maximum reached by ascending birds that is at least 2 ms^−1^ more supportive than the surface wind assistance WA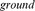 taken at 100 m AGL. Figure 2 illustrates the profiles of wind assistance (WA), wind (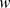), and bird density (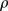) for the layering event on 19 May 2009 22:00 UTC. In this case, WA values were all negative due to an opposing side wind at all altitudes. At WA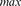 the winds were least prohibitive, i.e. the wind assistance optimum was in this case a wind hindrance minimum.

The identification of wind speed extrema was identical to that of wind assistance extrema. Low-Level Jets were identified following Baas et al. [34] as a maximum in the wind speed profile that is 2 ms^−1^ and 25% faster than the wind speed minimum directly above.

Using our definitions, layers and wind optima can occur between altitudes of 0.8–4.0 km, of which the lower range one may not considered truly ‘high altitude’. Nonetheless, we will refer to WA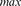 as a high-altitude wind optimum and refer to layers as high altitude migration. We use this terminology to distinguish such events from cases where wind assistance or bird density maxima are found near the ground (below 0.8 km).

## Results

**Figure 3:**
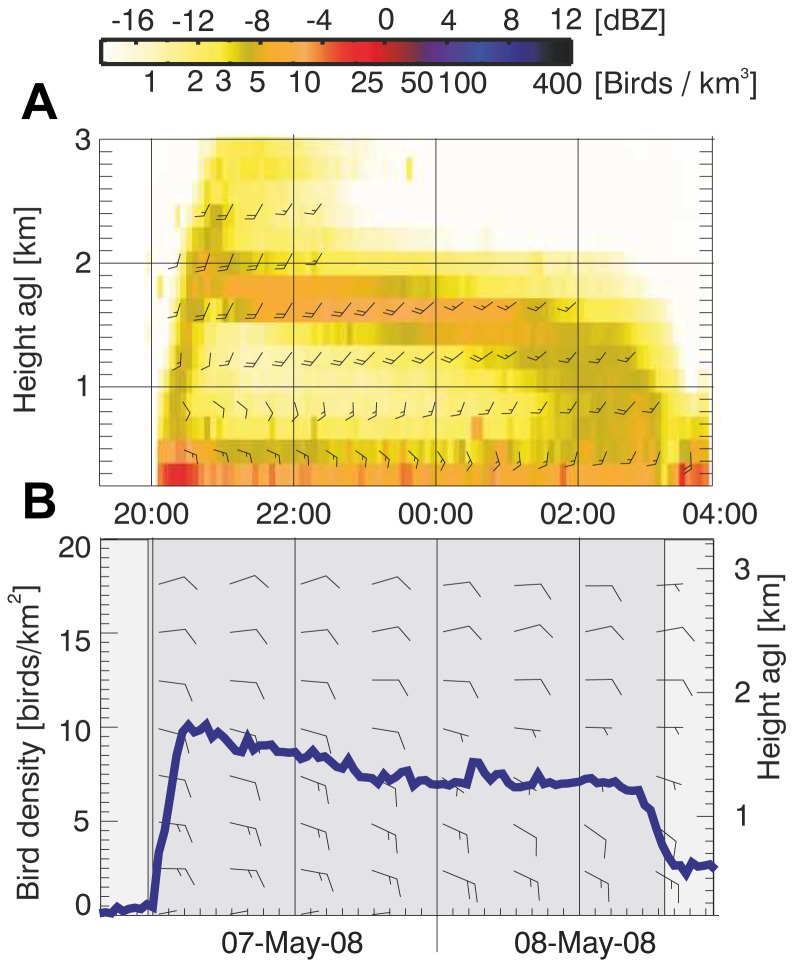
Example layering event. A: bird density profiles for the night of 7–8 May 2008, showing a layer at 1.8 km with north-easterly migrating birds. Strata below 500 m likely contain some scatterers that are traced by wind, such as insects, giving rise to deviating westerly flight directions. Flight speed and direction is indicated by barbs. B: corresponding altitude-integrated bird density (blue line, left axis) and wind profiles (wind barbs, right axis). In both panels, each half barb represents 10 km/hour and each full barb 20 km/hour.

An example of a typical mid-latitude layering event is shown in Figure 3. The top panel shows in colour the bird density as a function of time (horizontal axis) and altitude (vertical axis). A collective ascent at late dusk was followed by a steady layer that remains present for several hours at 1.8 km AGL. The bottom panel shows the wind speed and direction in terms of wind barbs (as well as the bird density integrated over height; blue line). In this case, wind speeds decreased with altitude, suggesting the layer may have formed to avoid opposing low-altitude cross wind. Layers may disappear during a collective descent at the end of the night, as seen in this example, though often gradually fade as birds stop migrating as the night progresses.

We observed 28 layering cases in spring 2008 and 16 layering cases in spring 2009. Layers occurred in 43% of the nights when rain was absent (n = 102). Therefore high altitude migration was relatively common in rain-free conditions in spring. In autumn, however, high altitude layering was rare, with only 2 identified events in two years.

**Figure 4:**
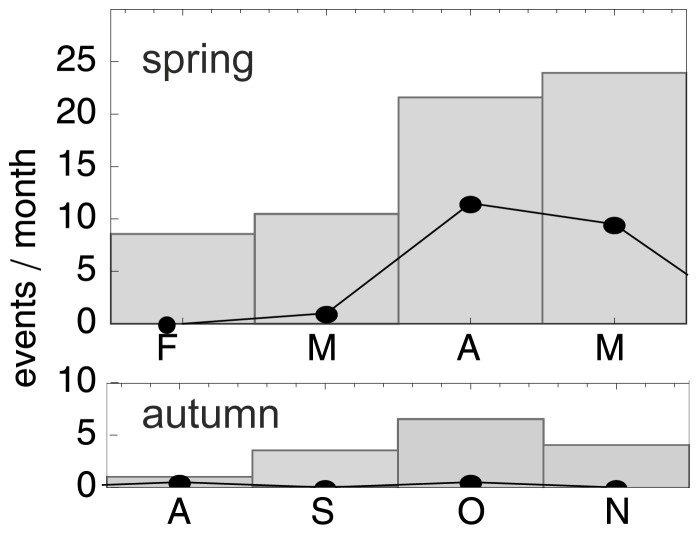
Seasonality. Top: Seasonal occurrence of migration layers (black dots and solid line) and high-altitude wind optima (bars) in spring (top) and autumn (bottom), 2008 and 2009. Both migration layers and high-altitude wind optima occur most frequently in spring in the months April and May.

Figure 4 shows the number of layer events specified per month (solid lines), as well as the mean number of high altitude wind optima (bars) for all nights in the study period (including nights with precipitation). It is clear that most layering events occurred in April and May, when also high altitude wind optima were present most frequently.

**Figure 5:**
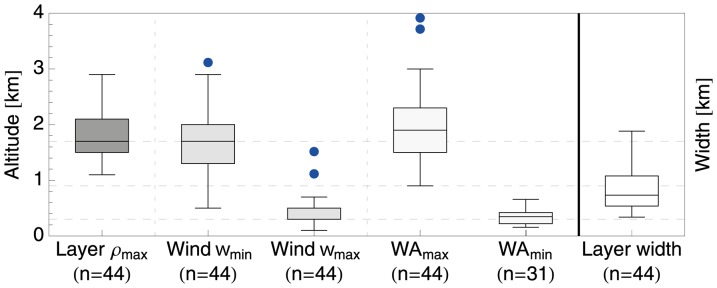
Observed height of migration layers 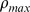
 **(dark grey) and their associated wind speed extrema** 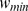 **,** 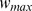 **(grey) and wind profit extrema WP** 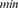 **and WP** 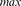 **, as defined in** **Figure 2A** **/B.** The migration layer width is displayed in the rightmost box (white).

We find that all layer events coincided with the presence of a high altitude wind optimum. Figure 5 summarises the altitudes of the layers, as well as the wind assistance and wind speed extrema during layering events, using the definitions of Figure 2 (see also Figure S2 of the supporting information for bird density profiles of all detected layering events). Layers formed at a mean altitude of 1.8
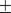
0.4 km, at similar altitude as the wind speed minimum 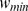 and wind assistance optimum WA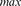. As illustrated in Figure 2C, the altitude of the optimum WA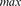 and layer altitude 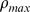 were clearly positively correlated (Fisher Ratio F = 33.4, p
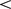
0.001, 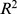 = 0.47). In 5 cases the wind profit profile was monotonically rising up to 4 km (indicated by open circles in Figure 2C) and other factors than wind likely limited flight altitude in these events. Notwithstanding, the presence of a high altitude wind optimum seems a prerequisite for layer formation, consistent with the idea that layers arise through the selection of high altitude winds that are more supportive (or less prohibitive) than surface winds.

**Figure 6:**
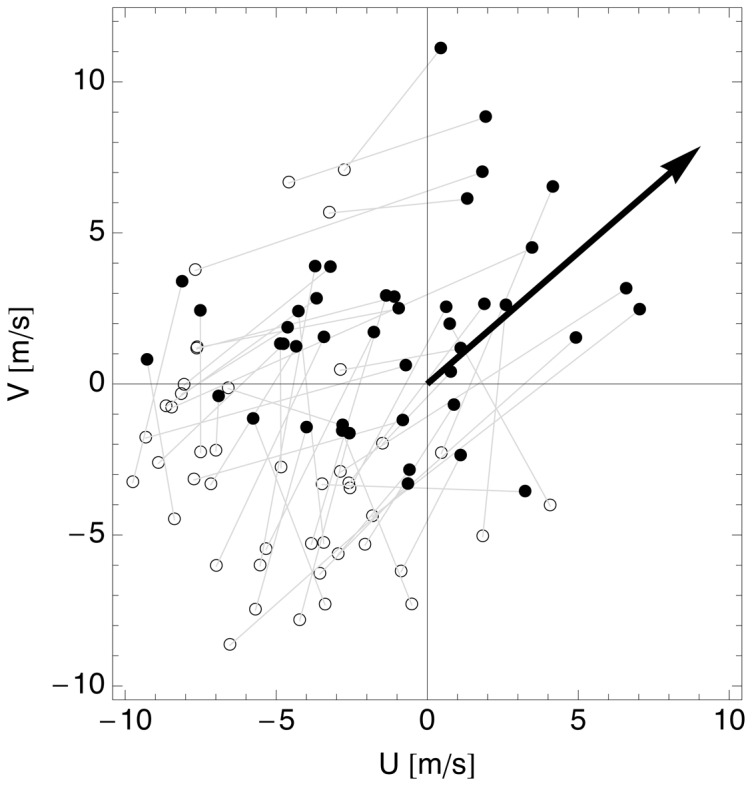
U and V components of the environmental wind near ground level (100 m AGL, open circles) and at the migration layer's altitude 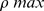
 **(filled arrow heads) for all layering events in spring 2008 and 2009.** The grey connecting lines represent the wind difference vector between the ground and layering strata. For comparison, the black solid arrow represents a bird's airspeed vector into the mean spring migratory direction (41°).

The large majority of wind profiles (95%) showed a well-developed low level jet [34]. Contrary to the circulation patterns found most commonly at mid-latitudes [1], [34], the highest wind speeds were thus found in the lower part of the atmosphere during layering events. In all but two cases surface wind opposed the migratory direction, resulting in negative wind assistance values near the ground (WA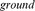 = −6
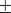
3 ms^−1^). Wind assistance values at at the layering altitude were on average close to zero (WA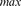 = 1
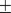
4 ms^−1^). The change in wind conditions achieved by the birds through ascending is further illustrated in Figure 6. The U and V components of both surface wind and wind at the layer height at 2.5 hours after sunset are shown, with corresponding surface and layer winds connected by grey lines. Surface winds were predominantly from the quadrant with winds from the NE, opposing the migratory direction (7.0
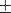
2.0 m/s, circular mean = 43°, circular variance = 0.25 at 100 m AGL), while layer winds were from variable directions (4.7
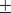
2.5 m/s, circular variance = 0.64) and weaker than surface winds (Mann-Whitney U, p
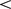
0.001). By migrating in high-altitude layers, birds thus effectively avoided unfavourable low altitude wind.

Synoptic weather charts during layering often showed a strong Omega blocking pattern above northern Europe, showing characteristic 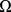-shaped 500 hPa geopotential height contours (see Figure S1 of the supporting information). Such large anticyclones redirect low-pressure cyclones (associated with unstable weather) towards its south-east and south-west, usually resulting in long-term stable weather conditions in spring and summer with high temperatures, clear skies and calm winds for up to a few weeks. The majority of layering events (32 cases) occurred within high pressure ridges, only 5 cases within a low-pressure trough and for 7 cases classification was difficult as migration took place in a pressure saddle point or intermediate region in between low and high pressure systems.

In summary, our results indicate that migration layers at our study site form by selection for wind (i.e. avoidance of prohibitive surface winds), predominantly in spring under influence of high pressure systems. We will use the meteorological framework introduced earlier to discuss to what extent this correlation between flight altitude, season and synoptic weather can be expected to be general.

## Discussion

**Figure 7:**
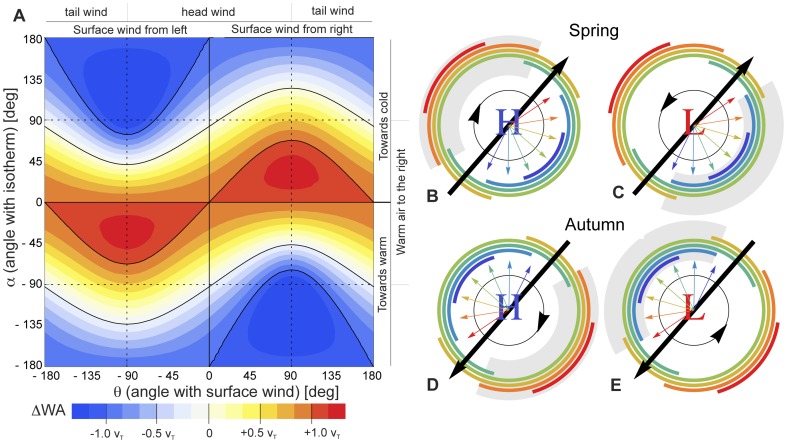
Altitudinal change in wind assistance in an ideal baroclinic atmosphere. A: Wind assistance change for a 1 km ascent in an ideal baroclinic atmosphere, as a function of surface wind angle 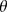 and isotherm angle 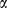 (see figure 1 for definition of angles). 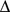
WA is calculated by filling out equation 2 into equation 3. We assume a bird's airspeed 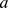 = 12 m/s, surface wind speed 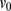 = 8 m/s, a surface temperature 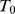 = 298 K and temperature gradient 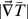 = 0.01 K/km, making 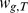 = 2.9 m/s according to equation 1. B–E: Hypothetical synoptic pressure systems in spring and autumn with a perfectly circular surface wind, blowing into the direction of the black circular arrow. Using the same parameters as in A, coloured arcs indicate angular sectors (i.e. geographic locations around the pressure system's centre), where a 1 km ascent results in substantial wind assistance improvement, which we define as 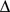
WA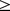.75 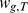. Each coloured arc corresponds to a different temperature gradient vector 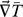 that may be present at a bird's location, drawn as an arrow in the same colour (arrows directed towards increasing temperature). The straight black arrow indicates the preferred migratory direction. The shaded sectors have positive wind assistance values already at the surface, denoting the regions with following surface winds.

In Figure 7A we show the wind assistance change 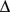
WA resulting from a bird's ascent of 1 km, as a function of its possible orientations with respect to surface wind direction and temperature gradient 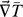. 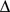
WA is calculated by filling out equation 2 into equation 3, under assumption of a horizontal synoptic scale change in temperature of 1 K per 100 km (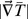 = 0.01 K/km). Red colours indicate more assisting winds with increasing altitude, while blue colours indicate more prohibitive winds with increasing altitude. From the figure it is evident that wind assistance increases strongly with altitude when a birds flies along an isotherm with warm air *to the right* (along or near the line 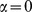
°). In this case the thermal wind is a pure tail wind. Conversely, birds flying along an isotherm with warm air *to the left* (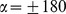
°) cannot improve wind assistance by ascending, since here the thermal wind is a pure head wind. Cross wind from the right may be reduced when flying along the temperature gradient towards cooler areas (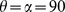
°) and cross winds from the left when flying towards warmer areas (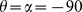
°). The fact that in our formulation of wind assistance the WA value improves with decreasing cross wind, gives rise to the bent asymmetric contours of 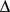
WA in Figure 7.

We may now identify the synoptic conditions and regions within cyclones and anticyclones where birds can take advantage of supportive winds at high altitude, by realising which synoptic temperature patterns occur frequently. First, as a result of the decrease in solar heating at higher latitudes, the north is generally cooler than the south and the large scale temperature gradient will have a southward component. Second, at sites under influence of a cool ocean the temperature gradient will have an inland component in summer and early autumn, which will be opposite in direction for easterly and westerly continental coasts.

Figure 7B–E depicts northern latitude high and low pressure systems in spring (B,C) and autumn (D,E) with a hypothetical circular flow around the synoptic centres. Coloured vectors indicate potential temperature gradients for which thermal wind effects improve the wind assistance at higher altitude in certain angular sectors surrounding the synoptic centres (temperature gradient vectors incapable of improving wind assistance have been omitted). Sectors where wind assistance improves considerably with altitude (which we define as 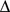
WA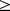.75 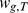) have been indicated by an arc of the same colour as its corresponding temperature gradient. For example, in the spring high pressure system of Figure 7B, when the large-scale temperature increases towards the south into the direction of the light-blue arrow, we expect improving wind assistance at higher altitude only in the light-blue angular sector, i.e. south-east of the pressure centre. Alternatively, when the large-scale temperature increases towards the north-east into the direction of the red arrow, winds improve at higher altitude only in the red angular sector, i.e. north-west of the pressure centre.

Whereas in spring the temperature gradients that can support favourable high altitude wind all point into typical directions for a westerly oceanic coast (east to south, Figure 7B/C), the temperature gradients in autumn point into uncommon directions (west to north, Figure 7D/E). Favourable conditions for high altitude migration will thus occur more often in spring than in autumn. This prediction is in line with the occurrence of high altitude migration layers as observed by radar in this study, found nearly exclusively in spring.

Interestingly, easterly coasts are predicted to facilitate more high-altitude wind optima in autumn than westerly coasts, since at easterly coasts the required westerly temperature gradient is more likely. This difference illustrates that opportunities for exploiting high altitude wind can vary between flyways. On the other hand, within specific flyways the altitudinal change of wind in synoptic systems shows predictable patterns, for example our prediction of generally favourable high altitude wind in easterly sectors of high pressure systems in western Europe. It is therefore conceivable that birds are aware of wind conditions higher in the atmosphere already at the ground, such that altitudinal wind condition can be factored into a bird's take-off decision.

Finally, the predicted synoptic patterns of high-altitude migration of Figure 7 highlights an important question: why were all layers detected in conditions with prohibitive surface winds and were hardly any layers detected in situations with following surface winds? For example, contrary to our predictions, no layers were observed in the south-eastern flank of low pressure systems in spring (grey-shaded area Figure 7C), although western Europe is frequently under influence of such a low pressure sector [35]. An explanation may lie in different altitude selection behaviour in opposing and following wind regimes. Birds encounter both regimes regularly and often need to migrate without tailwind assistance [4], [5]. High flight altitudes may occur primarily when birds have no alternative for migration at lower altitude due to low-level opposing winds, such as a low level jet in this mid-latitude study or an opposing trade in the tropics [12], [13]. As suggested earlier, birds may tend to avoid high wind speeds [36] and have a preference for low altitude migration in following wind conditions, even when wind assistance can be improved at higher altitude. Clearly, a deeper understanding of altitude selection by north temperate birds, including the effect of other factors besides wind, will be essential to fully reconstruct the synoptic patterns of flight altitude.

## Supporting Information

Figure S1
**Altitudinal change in wind assistance in an ideal baroclinic atmosphere.** Hirlam synoptic analysis for Europe for 8 May 2008 00 UTC. 500 hPa geopotential height is indicated in colors, surface pressure isobars are drawn in white. Our study site and the De Bilt radar are located at the blue cross in central Netherlands. The migration profile for this night is shown in Figure 3 of the original paper. All consecutive nights of the period 7–14 May 2008 showed formation of similar migration layers. The synoptic weather chart for this period showed a strong Omega blocking pattern above northern Europe, as can be seen from the characteristic 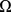-shaped 500 hPa geopotential height contours. Such large anticyclones effectively redirect low-pressure cyclones (associated with unstable weather) towards its south-east and south-west, usually resulting in long-term stable weather conditions in spring and summer with high temperatures, clear skies and calm winds.(PDF)

Figure S2
**Layering events in 2008 and 2009.** Profiles of bird density (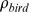 in birds/km^3^; red) and Wind Assistance (WA in m/s; green) for all detected layering events in the years 2008 and 2009. The horizontal red line indicates the wind optimum WA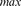. The horizontal green line indicates the layer altitude 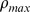.(PDF)

Text S1 **Thermal wind.** Derivation of the thermal wind equation.
(PDF)
