# Supplementary figures and images for: High Altitude Bird Migration at Temperate Latitudes: A Synoptic Perspective on Wind Assistance
Source: PLoS One. 2013 Jan 3;8(1):e52300. doi: 10.1371/journal.pone.0052300 (PMC3536796; doi:10.1371/journal.pone.0052300)

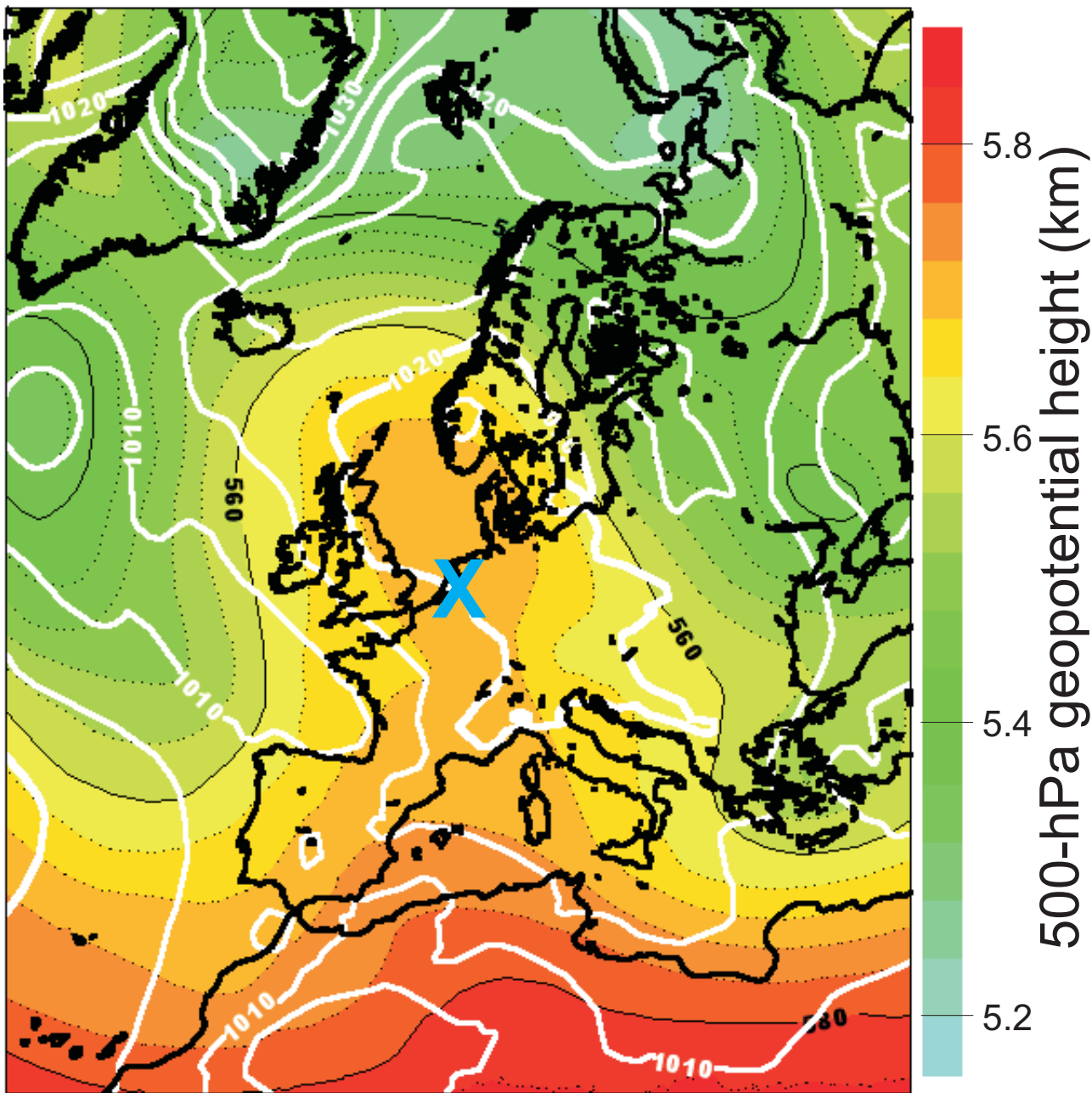

Supplement: Figure S1 — Altitudinal change in wind assistance in an ideal baroclinic atmosphere. Hirlam synoptic analysis for Europe for 8 May 2008 00 UTC. 500 hPa geopotential height is indicated in colors, surface pressure isobars are drawn in white. Our study site and the De Bilt radar are located at the blue cross in central Netherlands. The migration profile for this night is shown in Figure 3 of the original paper. All consecutive nights of the period 7–14 May 2008 showed formation of similar migration layers. The synoptic weather chart for this period showed a strong Omega blocking pattern above northern Europe, as can be seen from the characteristic -shaped 500 hPa geopotential height contours. Such large anticyclones effectively redirect low-pressure cyclones (associated with unstable weather) towards its south-east and south-west, usually resulting in long-term stable weather conditions in spring and summer with high temperatures, clear skies and calm winds. (PDF) [file pone.0052300.s001.pdf]

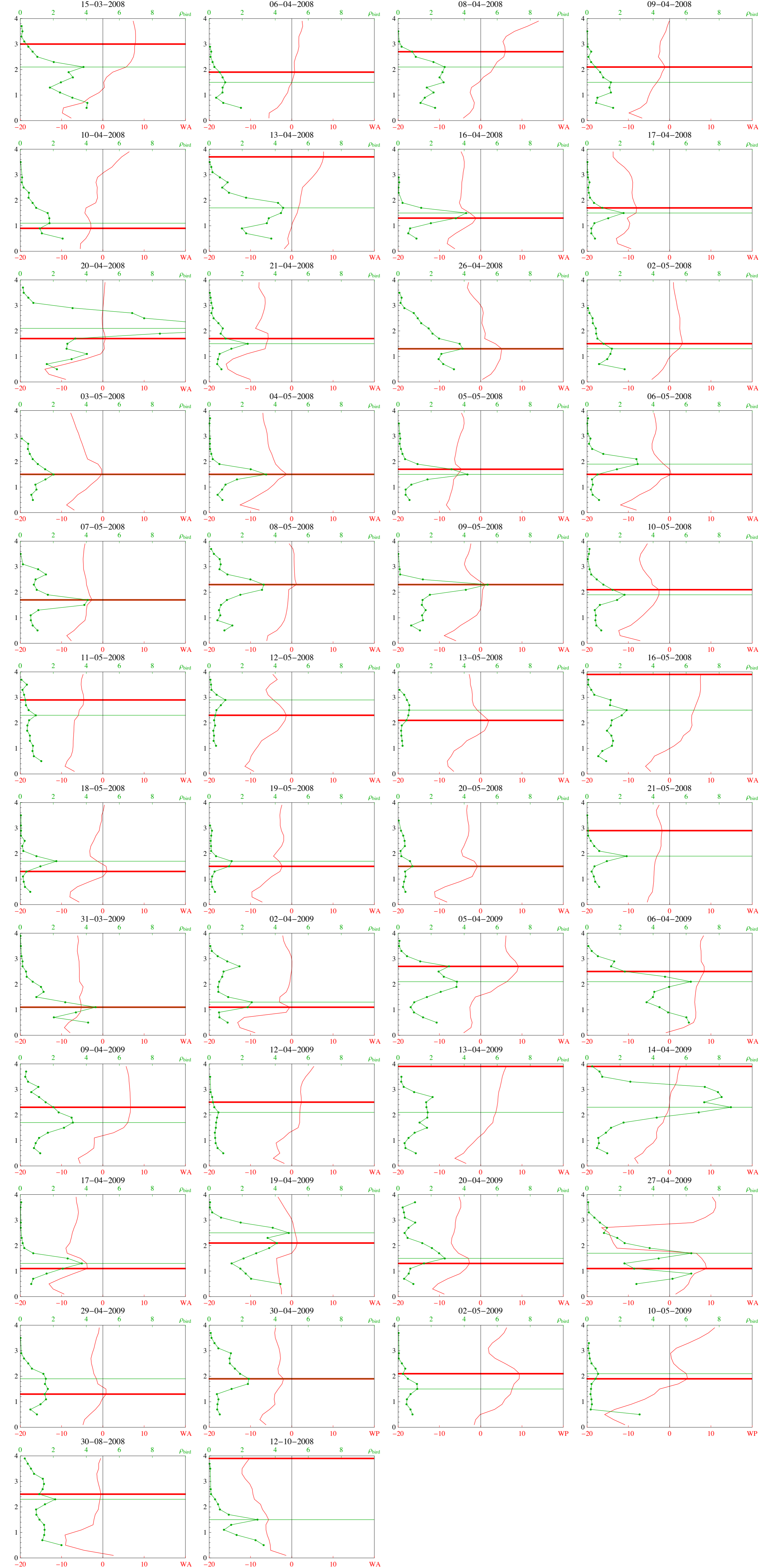

Supplement: Figure S2 — Layering events in 2008 and 2009. Profiles of bird density ( in birds/km3; red) and Wind Assistance (WA in m/s; green) for all detected layering events in the years 2008 and 2009. The horizontal red line indicates the wind optimum WA. The horizontal green line indicates the layer altitude . (PDF) [file pone.0052300.s002.pdf]
