# Supplementary material for: High Altitude Bird Migration at Temperate Latitudes: A Synoptic Perspective on Wind Assistance
Source: PLoS One. 2013 Jan 3;8(1):e52300. doi: 10.1371/journal.pone.0052300 (PMC3536796; doi:10.1371/journal.pone.0052300)
Supplement: Text S1 — Thermal wind. Derivation of the thermal wind equation. (PDF) [file pone.0052300.s003.pdf]

S1 thermal wind

For balanced geostrophic flow (for which friction with the earth's surface is neglected, curvature is small, and pressure gradient and Coriolis forces are in equilibrium), the geostrophic wind at a given pressure level p is given by

$$\vec{v}_g(p) = \frac{g}{f}\vec{k} \times \vec{\nabla}_p h, \tag{S1}$$

with g the gravitational constant, f the Coriolis parameter (approximately 10^{-4}/s at mid-latitudes), $\vec{k}$ the vertical unit vector and $\vec{\nabla}_p h$ the gradient in geopotential height at pressure level p (in the troposphere h is numerically almost identical to the height above mean sea level [1]). The geostrophic wind is a common approximation of the actual horizontal wind field for large-scale motions at mid-latitudes [1]. The vector outer product between $\vec{k}$ and $\vec{\nabla}_p h$ dictates that balanced flow is oriented along isobars, and the wind speed at a given pressure level is proportional to the spatial change in height of that level (i.e. when isobars are close together winds speeds are high).

The geostrophic wind difference between two altitudes h_0,h_1 ($h_0 < h_1$) is called the *thermal wind* $\vec{v}_{g,T} = \vec{v}_1 - \vec{v}_0$, which in an ideal gas atmosphere is given by [1]:

$$\vec{v}_{g,T} = \vec{k} \times \frac{g}{f}\vec{\nabla}_p(h_1 - h_0) = \frac{R}{fM}\vec{k} \times \vec{\nabla}\bar{T} \ln\left(\frac{p_0}{p_1}\right). \tag{S2}$$

$\vec{\nabla}\bar{T}$ is the gradient in the mean temperature of the air column between h_0 and h_1, M the molar mass of the earth's air and R the gas constant.

The above expression relates the vertical wind change within an air column to the large-scale patterns of surrounding temperature. The geostrophic thermal wind can be calculated explicitly if we know the vertical pressure buildup and temperature gradient at a given location. If we assume pressure is hydrostatic (given by the weight of air in the column above) a direct result of the ideal gas law is the so-called barometric formula, which expresses pressure as a function of altitude. For an atmosphere with constant environmental temperature lapse rate L, the barometric formula is given by

$$p(h) = P_0\left(\frac{T_0}{T_0 + Lh}\right)^{\frac{gM}{RL}} \tag{S3}$$

with h the geopotential height, T_0 the temperature at ground level and P_0 the pressure at ground level. We may now rewrite Eq. S2 by filling out the barometric formula Eq. S3 as

$$\vec{v}_{g,T}(h_1) = \frac{g}{fL}\vec{k} \times \vec{\nabla}\bar{T} \ln\left(1 + h_1 L/T_0\right) \approx \frac{gh_1}{fT_0}\vec{k} \times \vec{\nabla}\bar{T} \tag{S4}$$

where on the right-hand side the logarithm has been Taylor expanded up to first order in $h_1 L/T_0$, being a small number for h_1 up to a few kilometers. We emphasise that Eq. S4 should not be used to obtain quantitative estimates of wind, since atmospheric conditions are generally more complex (e.g. due to ageostrophic terms, moisture and turbulence). Our aim here however is only to provide qualitative insight in typical mid-latitude wind profiles.

References

1. Holton JR (2004) An introduction to dynamic meteorology, 4th edition. Academic Press.
